# Personalized warm-up strategies for adult athletes: a meta-analysis based on athletic level, gender, and region

**DOI:** 10.3389/fphys.2025.1706583

**Published:** 2025-12-04

**Authors:** Ye Xu, Jianmin Dai, Xingyue Liang, Yurou Zhang

**Affiliations:** Guangxi Zhuang Autonomous Region Sports Science Research Institute, Nanning, Guangxi, China

**Keywords:** parallel squat, post-activation potentiation (PAP), post-activation performance enhancement (PAPE), athletes, explosive power

## Abstract

**Objective:**

This study aimed to investigate the acute effects of high-intensity parallel squats (HIPS) on lower-limb explosive power in adult athletes, with a specific focus on how athletic calibre, sex and geographic origin modulate the ensuing potentiation response (PAP/PAPE)—the transient increase in muscular power or performance that follows heavy resistance exercise., and provide evidence for designing precision Warm-up protocols.

**Methods:**

Following PRISMA guidelines, 58 Randomized controlled trial (973 participants) published in six databases (Web of Science, PubMed, Cochrane, Embase, Scopus, and Ebsco) from 2004 to 2025 were systematically reviewed. Included studies utilized HIPS (≥85% 1RM) as a pre-activation stimulus, with countermovement jump (CMJ), standing long jump (SLJ), and short-distance sprints (10, 20, 30 m et al.) as outcome measures. Effect sizes were pooled using a random-effects model. Subgroup analyses based on athletic proficiency (high-level: squat 1RM/body weight ≥2), gender (male/female), and region (Asian/non-Asian) were conducted, with heterogeneity (I^2^ statistic) and publication bias (Egger’s test) assessed.

**Results:**

High-level athletes exhibited significant CMJ improvement after short and moderate intervals (p ≤ 0.05), whereas low-level athletes showed no gains and even transient inhibition post short intervals (p = 0.08). Non-Asian athletes demonstrated superior CMJ gains after long intervals (>8 min: WMD = 0.86, p = 0.01), while Asian athletes showed no improvement (p = 0.86). Males achieved moderate-interval CMJ enhancement (WMD = 0.95, p = 0.01), whereas females exhibited no significant changes (p = 0.64). In SLJ, low-level (WMD = 5.79, p = 0.01) and non-Asian athletes (WMD = 4.23, p = 0.02) showed gains, but sprint performance remained unaffected across subgroups (p > 0.05). Heterogeneity ranged from low to moderate (I^2^ = 0–70.6%).

**Conclusion:**

Athletes with high proficiency (squat 1RM/weight ≥2) can combine short/medium recovery intervals of HIPS warm-up to optimize vertical jump performance; athletes with low proficiency need to prioritize enhancing their basic strength before considering using HIPS for activation to improve acute exercise performance. Males are recommended to rest for 5–8 min after intervention activation and then proceed with training. Non-Asian athletes can attempt a long interval (>8 min) strategy.

## 1 Introduction

Explosive power is a critical determinant of athletic performance across most sports disciplines (Boullosa et al., 2013; Carlock et al., 2004), In individual sports such as jumping and combat events, the ability to maximize muscular power is essential for success (Wilson et al., 2013). Similarly, in team sports like rugby, volleyball, basketball, and soccer, lower limb explosive power directly correlates with sport-specific performance metrics, including acceleration, change-of-direction agility, and jumping capacity (Dobbs et al., 2019). Given its pivotal role, scientific evaluation of explosive power is imperative. In sports science, countermovement jump (CMJ), standing long jump (SLJ), and sprint performance (10–30 m) are widely adopted as key indicators for assessing explosive power (Brzycki, 1993; Mcmanus et al., 2018).

Adequate warm-up is a prerequisite for optimal explosive power output. Research suggests performance improvements arise not only from metabolic adaptations but also neuromuscular adjustments, such as the recruitment of high-threshold motor units (García-Pinillos et al., 2015). In the 1980s, studies observed that incorporating resistance training prior to power-based events acutely enhanced performance (Batista et al., 2011), leading to the concept of *postactivation potentiation (PAP)*. PAP refers to a transient increase in explosive power induced by submaximal resistance exercises, primarily attributed to enhanced neuromuscular conduction velocity (Meifu, 2018; Al Kitani et al., 2021), However, while PAP improves peak force and rate of force development (RFD), performance gains may not directly correlate with these physiological markers (Blazevich and Babault, 2019). For instance, some studies report performance enhancement without detectable PAP effects (Zimmermann et al., 2020). Due to limited direct evidence (e.g., myosin light-chain phosphorylation), PAP is hypothesized to originate from muscular rather than neural mechanisms (Blazevich and Babault, 2019; Lorenz, 2011), To reconcile discrepancies, Cuenca-Fernandez et al. proposed *post-activation performance enhancement (PAPE)* (Cuenca-Fe et al., 2017; Boullosa et al., 2020), which may involve residual PAP effects, increased muscle temperature (enhancing cross-bridge cycling via myosin ATPase activity (Stein et al., 1982))、altered fiber hydration (improving single cross-bridge efficiency (Sugi et al., 2015; Wang et al., 2015; Edman and Hwang, 1977)) and high-threshold motor unit recruitment (Blazevich and Babault, 2019; Elmubarak and Ranatunga, 1984). Given calcium ion (Ca^2+^) reuptake kinetics (half-life ≈28 s (Wilson et al., 2013; Vandervoort et al., 1983), PAP effects diminish within 4 min, while peak force enhancement typically occurs 6–10 min post-activation (Blazevich and Babault, 2019; Cuenca-Fe et al., 2017), Thus, performance gains beyond 4 min are attributed to PAPE (Blazevich and Babault, 2019; Cuenca-Fe et al., 2017; Xie et al., 2022; Docherty and Hodgson, 2007), now regarded as the primary mechanism for acute performance optimization (Zimmermann et al., 2020). Despite the established concept of PAE, the influence of moderating factors such as athletic level, gender, and regional background on the response to HIPS remains unclear and inconsistently reported, leading to heterogeneous recommendations.

Current research predominantly employs barbell squats as standardized pre-activation stimuli, yet the heterogeneity of PAE across gender, athletic proficiency, and regional demographics remains unclear (da Silva et al., 2024). Due to this heterogeneity, fatigue from activation means may exceed the potential postactivation effects if not properly intervened. It should be particularly emphasized that, based on the biological characteristics of human development, adolescent athletes—due to immature skeletal muscle development and high plasticity of neural regulatory mechanisms—require long-term motor skill learning when engaging in resistance training and should be trained with low-to-moderate intensity loads (Chaouachi et al., 2014; Faigenbaum et al., 2009). In contrast, adult athletes, possessing a fully developed neuromuscular system and metabolic adaptability, can tolerate high-intensity training stimulus (Wewege et al., 2017; Yamamoto et al., 2008).

This investigation categorizes resistance intensities as high- (≥85% 1RM), moderate- (60%–84% 1RM), and low-intensity (≤60% 1RM) (Wilson et al., 2013; Beato et al., 2021), with recovery intervals stratified into short (0–4 min), moderate (5–8 min), and long (≥8 min) durations (Xie et al., 2024; Seitz and Haff, 2016). This study collectively terms these mechanisms *postactivation effects (PAE)* to encapsulate their shared performance-enhancing properties. Focusing on high-intensity parallel squats (HIPS) as a pre-activation stimulus, we analyze time-dependent effects on lower limb explosive power (CMJ/SLJ) and sprint performance (10 m, 20 m, 30 m et al.) across three dimensions: gender (male/female), athletic proficiency (high/low: squat 1RM/body weight ≥2 vs. <2), and regional characteristics (Asian/non-Asian). We hypothesized that high-level, male, and non-Asian athletes would demonstrate a greater and more rapid PAE response to HIPS. The goal is to establish population-specific PAE application models, refining precision in training protocols.

## 2 Methods

### 2.1 Protocol and registration

This systematic review and meta-analysis strictly adhered to the Preferred Reporting Items for Systematic Reviews and Meta-Analyses (PRISMA) guidelines (Page et al., 2021), The protocol was registered in PROSPERO (Registration ID: CRD420251002084).

### 2.2 Search strategy and study selection

A comprehensive literature search was conducted across six databases (Web of Science, PubMed, Cochrane, Embase, Scopus, and Ebsco) to identify Randomized controlled trial (RCT) investigating the effects of HIPS on lower limb jump and sprint performance in adult athletes. The search timeframe spanned from database inception to 4 July 2025. Key search terms included Medical Subject Headings (MeSH) and free-text keywords such as “Squat,” “Post-activation Potentiation,” “Post-activation Performance Enhancement,” “Athletes,” and “Explosive Power.”

Three independent reviewers conducted literature searches and screened eligible studies. Discrepancies were resolved through consultation with a fourth reviewer. Additionally, reference lists of included studies and relevant systematic reviews were cross-checked to identify potentially eligible trials.

### 2.3 Eligibility criteria

Studies were evaluated using the PICOS framework (Participants, Intervention, Comparator, Outcomes, Study Design) (Liberati et al., 2009).

- P: Adult athletes (Age ≥18)
- I: High-intensity parallel squat (HIPS)
- O: Explosive power metrics
- S: Randomized controlled trial (RCT)

Studies meeting all criteria below were included:

#### 2.3.1 Population

Studies recruiting adult athletes aged ≥18 years, stratified by predefined variables:

- Gender (male/female)
- Region (Asian/non-Asian)
- Athletic proficiency (high-level: squat 1RM/body weight ≥2; low-level: squat 1RM/body weight <2) (Suchomel et al., 2016).

#### 2.3.2 Intervention

Pre-activation intervention: High-intensity parallel squat (HIPS).

#### 2.3.3 Comparator

Single-group design with pre- and post-intervention measurements (baseline vs. post-HIPS).

#### 2.3.4 Outcome

Studies reporting at least one outcome:

- Countermovement jump (CMJ)
- Standing long jump (SLJ)
- Sprint performance (10 m, 20 m, 30 m et al.)

#### 2.3.5 Study design

Randomized controlled trial (RCT).

### 2.4 Exclusion criteria

Studies were excluded if they met any criterion below:

1. No reported participant age or inclusion of individuals <18 years.
2. Non-athlete population
3. Interventions not involving HIPS (failure to achieve femoral parallel alignment at the lowest squat position or intensity <85% 1RM).
4. Non-peer-reviewed literature (e.g., dissertations, protocols, conference abstracts, gray literature).
5. Insufficient data for analysis.

### 2.5 Data extraction

A predefined extraction form was used to collect:

- Study characteristics (first author, publication year)
- Population details (age, gender, region, athletic proficiency, sample size)
- Intervention parameters (type, post-test timing)
- Outcome metrics

Missing data were requested via email (three attempts over 3 weeks) from corresponding authors. Data extraction was independently performed by two reviewers and verified by a third. Disagreements were resolved through consensus.

### 2.6 Measures of treatment effect

Intervention effects were assessed using mean difference (MD) and standard deviation (SD) of pre-post changes. For studies lacking SD values, estimates were derived from standard error (SE), 95% confidence interval (CI), *p*-values, or *t*-statistics (Chandler et al., 2019). A correlation coefficient of 0.5 was assumed for SD calculations, reflecting moderate measurement consistency and balancing variability between pre- and post-intervention assessments (Chandler et al., 2019).

### 2.7 Quality assessment of evidence

#### 2.7.1 Risk of bias

Evaluated via the Cochrane Risk of Bias Tool (version 2.0) across domains: randomization, allocation concealment, blinding, incomplete outcome data, and selective reporting (Ste et al., 2019). Studies were classified as:

- *Low risk*: All domains low risk
- *High risk*: ≥1 domain high risk
- *Some concerns*: Unclear risk

#### 2.7.2 Evidence certainty

Assessed using the GRADE framework via GRADEpro GDT (www.gradepro.org). The GRADE ratings were conducted by two independent reviewers (YX and JMD). In case of disagreement, the issue was resolved through consultation or by a third researcher (XY L). Outcomes were graded as “high,” “moderate,” “low,” or “very low” based on risk of bias, inconsistency, indirectness, imprecision, and publication bias (Guyatt et al., 2011).

### 2.8 Statistical analysis

- Meta-analysis was conducted for outcomes reported in ≥2 homogeneous studies. Effects were quantified as: (Guyatt et al., 2011): CMJ/SLJ: Weighted mean difference (WMD) with 95% CI
- Sprint performance: Standardized mean difference (SMD) with 95% CI

A random-effects model addressed heterogeneity across populations, interventions, and measurement protocols (Deeks et al., 2019). Heterogeneity was assessed via *I* ^2^ statistics:

• <25%: Low
• 25%–75%: Moderate
• 75%: High (Higgins and Thompson, 2002).

Publication bias was evaluated via Egger’s test (Egger et al., 1997). If detected,the trim-and-fill method adjusted effect estimates (Duval and Trim, 2000).

Subgroup analyses explored heterogeneity by recovery intervals (short: 0–4 min; moderate: 5–8 min; long: ≥8 min) (Xie et al., 2024; Seitz and Haff, 2016). Sensitivity analyses identified outlier studies influencing high heterogeneity. All statistical analyses were performed using the metan package within Stata software (version 17.0; StataCorp, College Station, TX, USA), with forest plots visualizing pooled effects. Statistical significance was set at *p* < 0.05.

## 3 Results

### 3.1 Literature selection and study characteristics

**FIGURE 1:**
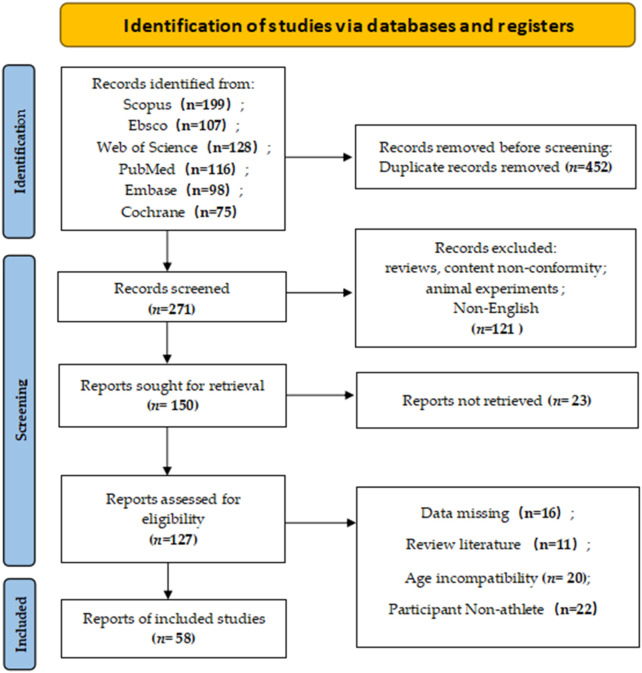
PRISMA Flow diagram of the search process for studies.

A total of 723 records were retrieved from six databases: Web of Science (n = 128), PubMed (n = 116), Cochrane (n = 75), Embase (n = 98), Scopus (n = 199), and Ebsco (n = 107). After removing duplicates using EndNote X9, 271 records remained. Screening titles and abstracts excluded 121 irrelevant studies, leaving 150 records for full-text review. Following full-text assessment, 23 studies were excluded due to ineligibility, resulting in 127 studies. Ultimately, 58 studies were included for analysis. The study selection process is illustrated in Figure 1. All included studies were published between 2004 and 2024, with varying sample sizes, durations, and intervention protocols. The 58 studies comprised 67 experimental groups (973 participants). Among these, 49 studies reported countermovement jump (CMJ) outcomes, 3 reported standing long jump (SLJ), and 12 reported sprint performance (10 m, 20 m, 30 m). Detailed characteristics of the included studies are presented in Table 1.

**TABLE 1:** Basic characteristics of the included studies.

Serial	Author (Year)	Load (Sets × Reps × %1RM)	Intervals (min)	Gender	Region
1	Shi et al. (2023)	1% × 3% × 85%	0.5, 3, 6, 9	Male	Asia
2	Atalag et al. (2021)	1% × 3% × 90%	3	All	Other
3	Matth et al. (2004)	1% × 5% × 85%	10	Male	Other
4	Bauer et al. (2018)	1% × 5% × 60%; 1% × 4% × 90%	0.25–11	Male	Asia
5	Krzysztofik et al. (2023a)	3% × 3% × 85%	3, 6, 9	Male	Other
6	Atalağ et al. (2020)	1% × 3% × 90%	8	All	Other
7	Krzysztofik et al. (2023b)	1% × 2% × 60%; 3% × 3% × 85%	2–10	Male	Other
8	Boyd et al. (2014)	1% × 3% × 90%	2, 5, 8, 11	Male	Other
9	Chen et al. (2024)	1% × 3% × 93%	0.25–20	Male	Other
10	Carbone et al. (2020)	3% × 3% × 85%	8	Male	Other
11	Moir et al. (2011)	1% × 12% × 37%; 1% × 3% × 90%	2	Female	Asia
12	Urbański et al. (2023)	1% × 3% × 90%	5	Unknow	Other
13	Crum et al. (2012)	3% × 1% × 50%; 3% × 1% × 65%	3,5,10,15	Male	Other
14	Nickerson et al. (2018)	1% × 3% × 85%	1,4,7,10	Male	Other
15	Pálinkás et al. (2024)	5% × 3% × 85%	0	Male	Other
16	Pálinkás et al. (2024)	5% × 3% × 85%	0	Female	Other
17	Yuan et al. (2023)	2% × 5% × 85%	4,8,12,16	Male	Asia
18	Do Carmo et al. (2021)	1% × 5% × 85%	4	Male	Other
19	Crewther et al. (2011)	1% × 3% × 90%	4,8,12,16	Male	Other
20	Khamoui et al. (2009)	5% × 3% × 85%	5	Male	Other
21	Faller et al. (2023)	1% × 3% × 90%	0.75	Male	Other
22	Seitz et al. (2014b)	1% × 3% × 90%	7	Unknow	Other
23	Fletcher (2013)	1% × 2% × 90%	4	Male	Other
24	Bielitzki et al. (2021)	1% × 3% × 91%	8	Male	Other
25	Comyns et al. (2010)	1% × 3% × 91%	4	Male	Other
26	Esformes and Bampouras (2013)	1% × 3% × 91%	5	Male	Other
27	Lowery et al. (2012)	Multi-load	0–12	Male	Other
28	Heynen et al. (2024)	3% × 3% × 85%	2 min, 2 h	Female	Other
29	Mccann and Flanagan (2010)	1% × 5% × 85%	4,5	All	Other
30	Hornikel et al. (2023)	1% × 4% × 75%	4	Unknow	Other
31	Lim and Kong (2013)	1% × 3% × 90%	4	Male	Other
32	Bevan et al. (2010)	1% × 3% × 91%	8	Male	Other
33	da Silva et al. (2024)	3% × 3% × 90%	6	Female	Other
34	da Silva et al. (2024)	5% × 3% × 85%	0	Male	Other
35	Hughes et al. (2016)	1% × 3% × 91%	6	Male	Other
36	Kolinger et al. (2024)	1% × 3% × 90%	5,8,11	Male	Other
37	Villalon-Gasch et al. (2022)	1% × 3% × 90%	8	Unknow	Asia
38	West et al. (2013)	1% × 3% × 87%	8	Unknow	Other
39	Jirovska et al. (2023)	1% × 3% × 85%	0.5,4,8,12	Male	Other
40	Márquez et al. (2023)	1% × 3% × 80%	1,4	Male	Other
41	Marin et al. (2021)	3% × 3% × 85%	2,4,6,8	Male	Other
42	Sañudo et al. (2020)	1% × 3% × 90%	4	Unknow	Other
43	Evetovich et al. (2015)	1% × 3% × 80%	8	All	Other
44	Evetovich et al. (2015)	1% × 3% × 80%	8	Male	Other
45	Montalvo et al. (2021)	1% × 5% × 85%	3	All	Other
46	Nibali et al. (2015)	1% × 5% × 85%	4,8,12	Male	Other
47	Nickerson et al. (2019)	1% × 3% × 85%	1,4,7,10	Male	Other
48	Hester et al. (2017)	1% × 5% × 80%	1,3,5,10	Male	Other
49	Piper et al. (2020)	3% × 5% × 87%	0.33–20	Male	Other
50	Mina et al. (2019)	1% × 3% × 85%	0.5,4,8,12	Male	Other
51	Scott and Docherty (2004)	1% × 5% × 85%	5	Male	Other
52	Shi et al. (2024)	5% × 1% × 90%	0,4,8,12	Male	Asia
53	Suchomel et al. (2016)	1% × 2% × 90%	1–10	Male	Other
54	Ah Sue et al. (2016)	1% × 5% × 85%	2,6,10,14,18	Female	Other
55	Villalon-Gasch et al. (2020)	1% × 3% × 90%	8	Unknow	Other
56	Yen Yeh et al. (2024)	3% × 3% × 85%	0,2,4,6	All	Other
57	Zheng et al. (2024)	1% × 15% × 30%	0,3,6,9,12	Male	Asia
58	Wen-Xia et al. (2018)	1% × 3% × 90%	3,6,9,12	Male	Asia

### 3.2 Risk of bias, certainty of evidence

**FIGURE 2:**
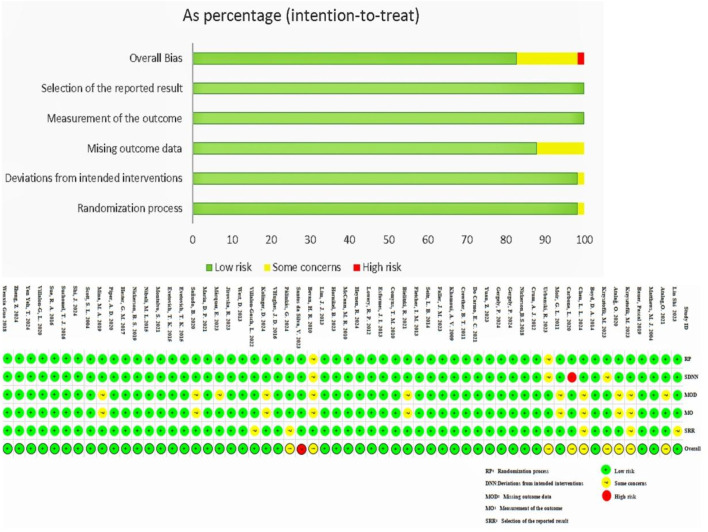
Overall risk of bias presented as percentage of each risk of bias item across all included studies. Green = Low risk, Red = High risk, Yellow = Some concerns.

The risk of bias assessment is presented in Figure 2. Overall, 48 studies (82.8%) were classified as having a *low risk of bias*, 9 studies (15.5%) were classified as having *some concerns*, and 1 study (1.7%) was classified as having a *high risk of bias* (Figure 2).

Regarding the randomization process, 57 studies (98.3%) explicitly described the method of random sequence generation. Only 1 study (1.7%) failed to report allocation concealment, while all others provided sufficient information, resulting in a low risk of bias for most studies in this domain.

For bias due to missing outcome data, 12 studies (12.1%) did not report raw data directly but presented results graphically, leading to a classification of *some concerns*. The remaining studies were assessed as having a *low risk of bias* in this domain.

In the domain of *other biases*, no additional sources of bias were identified across all studies, and thus, all were rated as having a *low risk of bias*.

### 3.3 Results of individual studies

Table 2 shows the results of the acute effects of HIPS on the explosive power of adult athletes.

**TABLE 2:** Summary of all analysis results.

Subject classification standard		Index	Intermittent time (min)							
			Totality		0–4min		5–8min		>8min	
			p	I^2^ (%)	p	I^2^ (%)	p	I^2^ (%)	p	I^2^ (%)
Level	High	CMJ	0.00^a^	0	p < 0.05	0	p < 0.05	0	p > 0.05	0
	Low	CMJ	0.47	37.7	p > 0.05	61.3	p > 0.05	0	p > 0.05	0
		SLJ	0.01^a^	0	—	—	—	—	—	—
		Sprint	0.61	0	p > 0.05	0	p > 0.05	0	p > 0.05	0
District	Asia	CMJ	0.86	0	p > 0.05	38.2	p > 0.05	0	p > 0.05	0
	Non-Asian	CMJ	0.54	59.8	p > 0.05	70.6	p > 0.05	40	p < 0.05	29
		SLJ	0.02^a^	0	—	—	—	—	—	—
		Sprint	0.36	0	p > 0.05	0	p > 0.05	0	p > 0.05	0
Gender	Male	CMJ	0.65	0	p > 0.05	60.2	p < 0.05	0	p > 0.05	0
		Sprint	0.47	0	p > 0.05	0	p > 0.05	0	p > 0.05	0
	Female	CMJ	0.67	70.6	—	—	—	—	—	—

^a^ Indicates statistically significant difference.

#### 3.3.1 Effects of HIPS on explosive power in high- and low-level adult athletes

##### 3.3.1.1 Vertical jump performance

###### 3.3.1.1.1 High-level athletes

**FIGURE 3:**
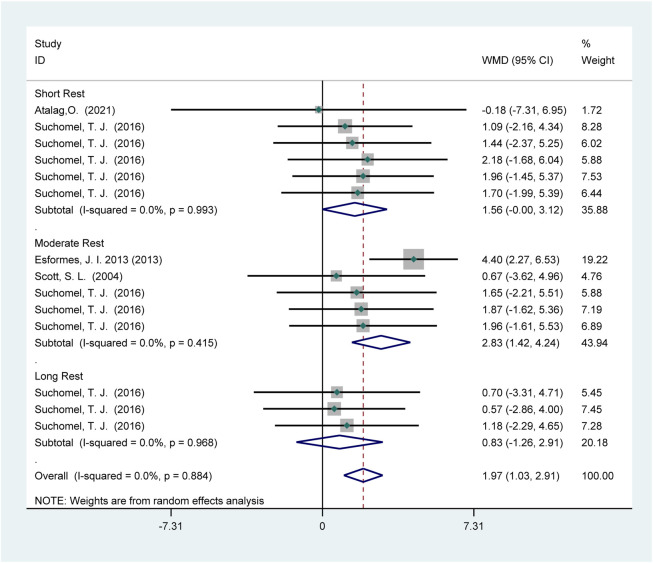
Effects of HIPS on CMJ of high-level adult athletes. Note: The diamond represents the pooled effect size; its center corresponds to the point estimate of the pooled effect, and its width reflects the 95% confidence interval (CI). The CI indicates the uncertainty around the effect size; if it does not overlap the null line, the effect is considered statistically significant.

Four studies (69 participants) reported the effects of HIPS on vertical jump performance (Figure 3). Meta-analysis revealed a significant improvement in countermovement jump (CMJ) performance (WMD = 1.97; 95% CI: 1.03 to 2.91; *p* = 0.00). Subgroup analysis showed significant CMJ improvements after short (WMD = 1.56; 95% CI: −0.00 to 3.12; *p* = 0.05) and moderate recovery intervals (WMD = 2.83; 95% CI: 1.42 to 4.42; *p* = 0.00), but no significant improvement after long intervals (WMD = 0.83; 95% CI: −1.26 to 2.91; *p* = 0.44). Heterogeneity was absent (*I* ^2^ = 0%).

###### 3.3.1.1.2 Low-level athletes

**FIGURE 4:**
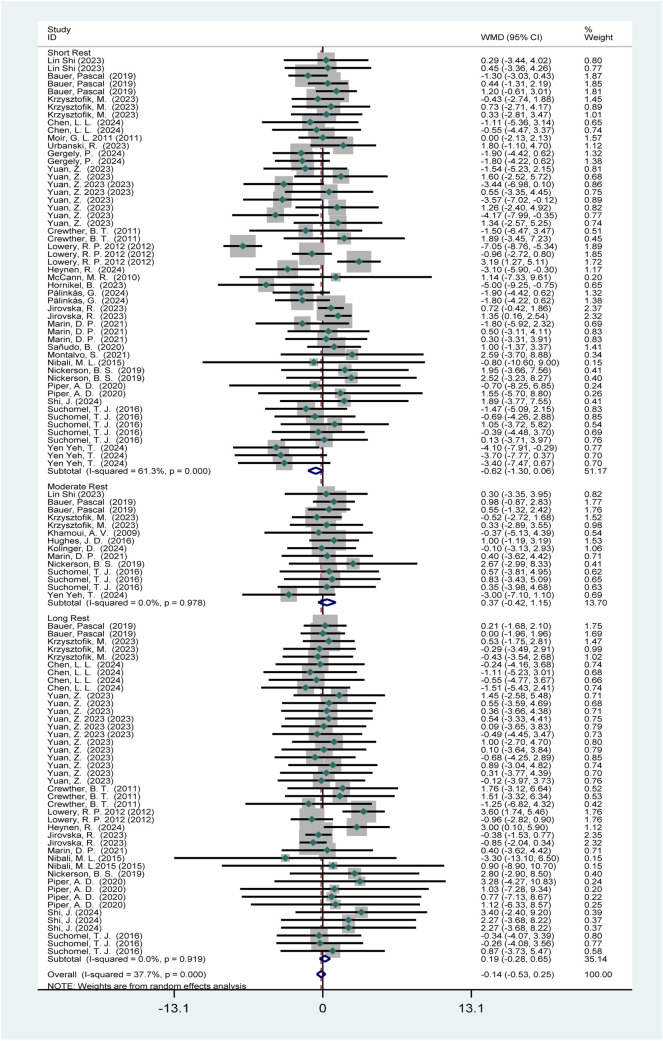
The effect of HIPS on CMJ of low level adult athletes.

Thirty-three studies (568 participants) reported the effects of HIPS on vertical jump performance (Figure 4). Meta-analysis showed no significant improvement in CMJ performance (WMD = −0.14; 95% CI: −0.53 to 0.25; *p* = 0.47). Subgroup analysis indicated no significant improvements after moderate (WMD = 0.37; 95% CI: −0.28 to 0.65; *p* = 0.36) or long intervals (WMD = 0.19; 95% CI: −1.26 to 2.91; *p* = 0.43). A near-significant trend (*p* = 0.08) suggested potential inhibition after short intervals (WMD = −0.62; 95% CI: −0.42–1.15). Moderate heterogeneity was observed (*I* ^2^ = 37.7%).

##### 3.3.1.2 Horizontal jump performance

###### 3.3.1.2.1 Low-level athletes

**FIGURE 5:**
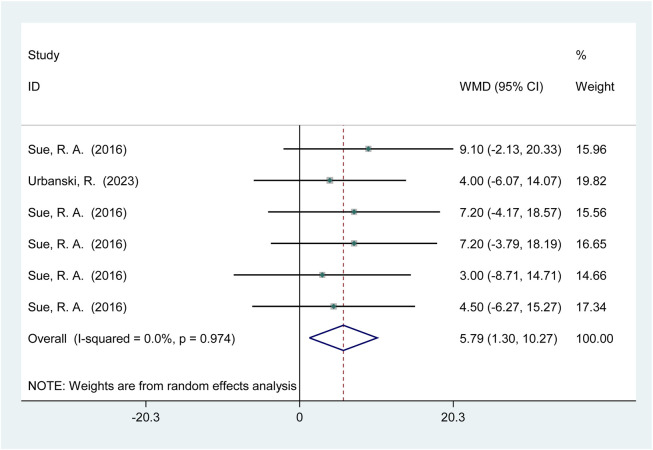
Effects of HIPS on SLJ of low level adult athletes.

Two studies (26 participants) reported the effects of HIPS on horizontal jump performance (Figure 5). Meta-analysis showed a significant improvement in standing long jump (SLJ) performance (WMD = 5.79; 95% CI: 1.30 to 10.27; *p* = 0.01). No heterogeneity was observed (*I* ^2^ = 0%).

##### 3.3.1.3 Sprint performance

###### 3.3.1.3.1 Low-level athletes

**FIGURE 6:**
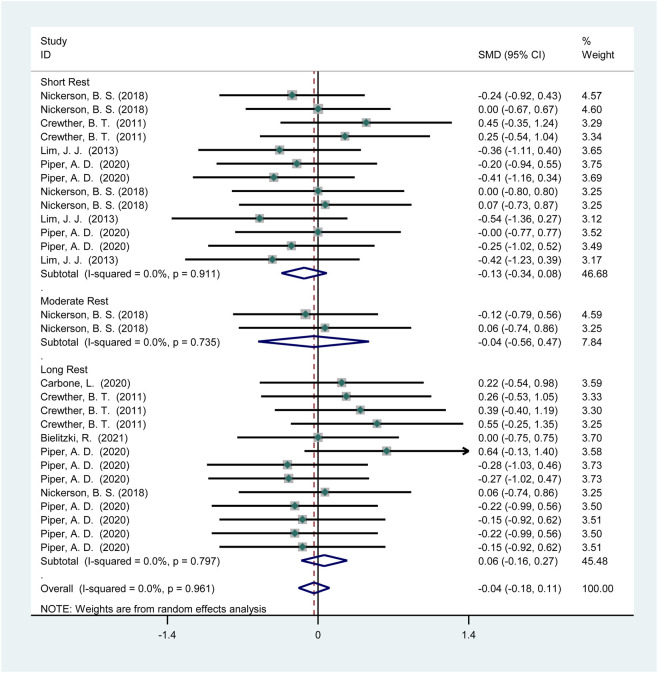
The effect of HIPS on sprint ability of low level adult athletes.

Seven studies (115 participants) reported the effects of HIPS on sprint performance (Figure 6). Meta-analysis revealed no significant improvement in sprint performance (SMD = −0.04; 95% CI: −0.18 to 0.11; *p* = 0.61). Subgroup analysis showed no significant improvements after short (SMD = −0.13; 95% CI: −0.34 to 0.08; *p* = 0.23), moderate (SMD = −0.04; 95% CI: −0.56 to 0.47; *p* = 0.88), or long intervals (SMD = 0.06; 95% CI: −0.16 to 0.27; *p* = 0.61). No heterogeneity was observed (*I* ^2^ = 0%).

#### 3.3.2 Effects of HIPS on explosive power in Asian and Non-Asian adult athletes

##### 3.3.2.1 Vertical jump performance

###### 3.3.2.1.1 Asian athletes

**FIGURE 7:**
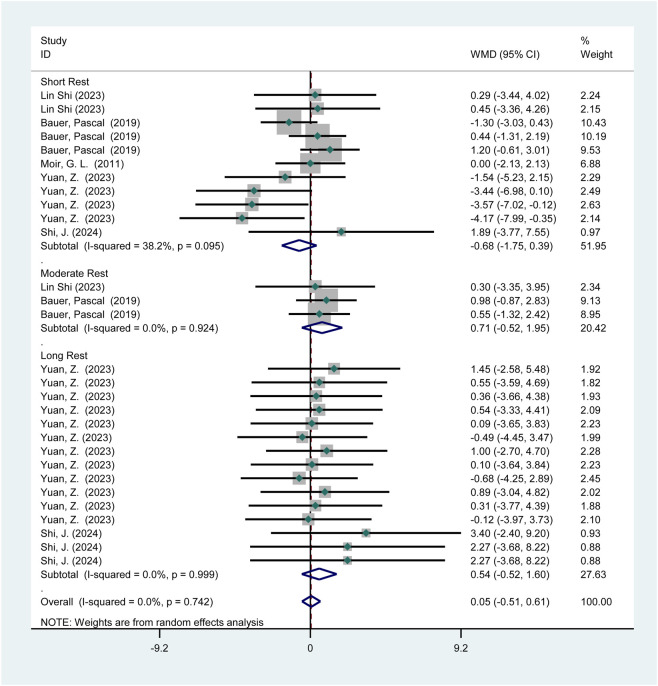
The effect of HIPS on CMJ of Asian adult athletes.

Five studies (119 participants) reported the effects of HIPS on vertical jump performance (Figure 7). Meta-analysis showed no significant improvement in CMJ performance (WMD = 0.05; 95% CI: −0.51 to 0.61; *p* = 0.86). Subgroup analysis indicated no significant improvements after short (WMD = −0.68; 95% CI: −1.75 to 0.39; *p* = 0.21), moderate (WMD = 0.71; 95% CI: −0.52 to 1.95; *p* = 0.26), or long intervals (WMD = 0.54; 95% CI: −0.52 to 1.60; *p* = 0.32). No heterogeneity was observed (*I* ^2^ = 0%).

###### 3.3.2.1.2 Non-Asian athletes

**FIGURE 8:**
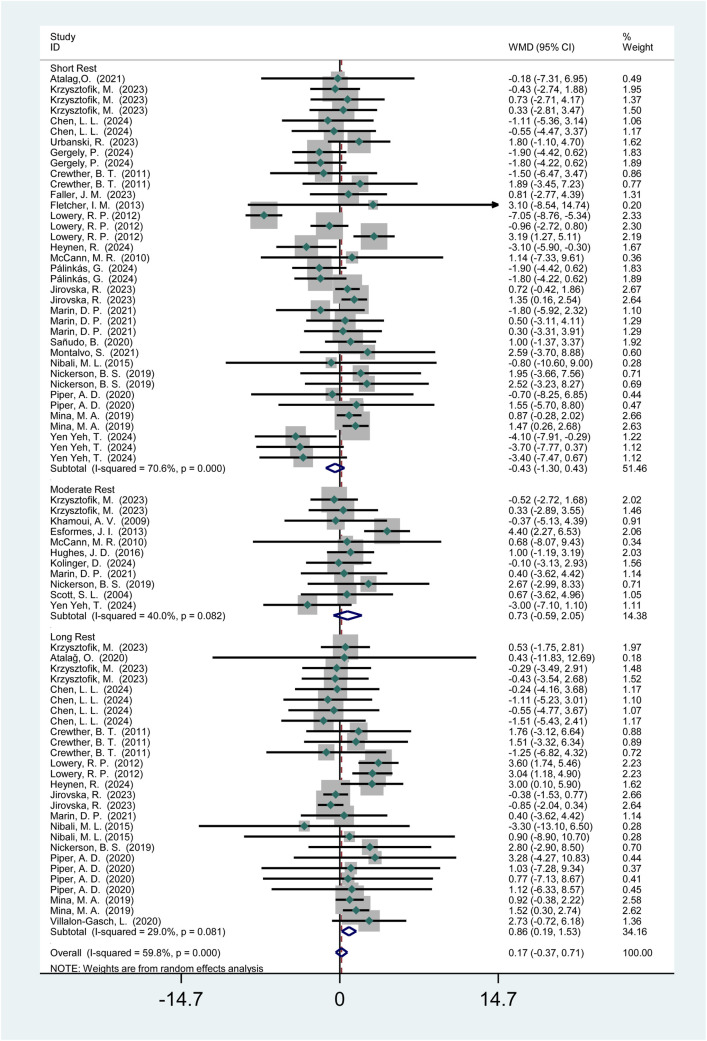
Effects of HIPS on CMJ of non-Asian adult athletes.

Twenty-seven studies (436 participants) reported the effects of HIPS on vertical jump performance (Figure 8). Meta-analysis showed no significant improvement in CMJ performance (WMD = 0.17; 95% CI: −0.45 to 0.62; *p* = 0.54). Subgroup analysis indicated no significant improvements after short (WMD = −0.43; 95% CI: −1.30 to 0.43; *p* = 0.33) or moderate intervals (WMD = 0.73; 95% CI: −0.59 to 2.05; *p* = 0.28), but a significant improvement after long intervals (WMD = 0.86; 95% CI: 0.07 to 1.17; *p* = 0.01). Moderate heterogeneity was observed (*I* ^2^ = 45.9%).

##### 3.3.2.2 Horizontal jump performance

###### 3.3.2.2.1 Non-Asian athletes

**FIGURE 9:**
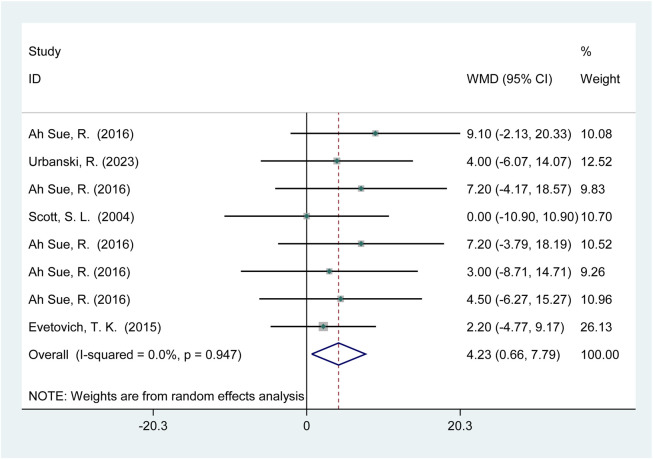
Effects of HIPS on SLJ of non-Asian adult athletes.

Four studies (60 participants) reported the effects of HIPS on horizontal jump performance (Figure 9). Meta-analysis showed a significant improvement in SLJ performance (WMD = 4.23; 95% CI: 0.66 to 7.79; *p* = 0.02). No heterogeneity was observed (*I* ^2^ = 0%).

##### 3.3.2.3 Sprint performance

###### 3.3.2.3.1 Non-Asian athletes

**FIGURE 10:**
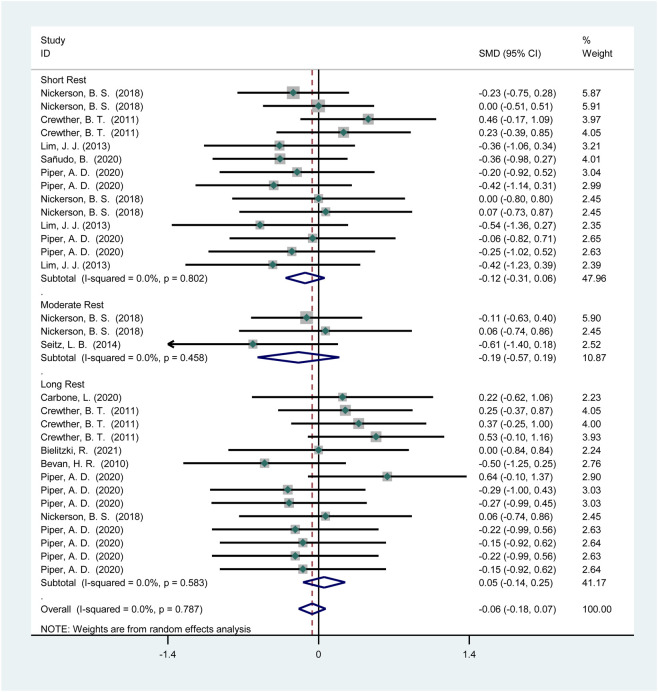
The effect of HIPS on sprint ability of non-Asian adult athletes.

Twelve studies (186 participants) reported the effects of HIPS on sprint performance (Figure 10). Meta-analysis revealed no significant improvement in sprint performance (SMD = −0.06; 95% CI: −0.18 to 0.07; *p* = 0.36). Subgroup analysis showed no significant improvements after short (SMD = −0.12; 95% CI: −0.31 to 0.06; *p* = 0.19), moderate (SMD = −0.19; 95% CI: −0.57 to 0.19; *p* = 0.34), or long intervals (SMD = 0.05; 95% CI: −0.14 to 0.25; *p* = 0.61). No heterogeneity was observed (*I* ^2^ = 0%).

#### 3.3.3 HIPS effects of HIPS on explosive power in male and female adult athletes

##### 3.3.3.1 Vertical jump performance

###### 3.3.3.1.1 Male athletes

**FIGURE 11:**
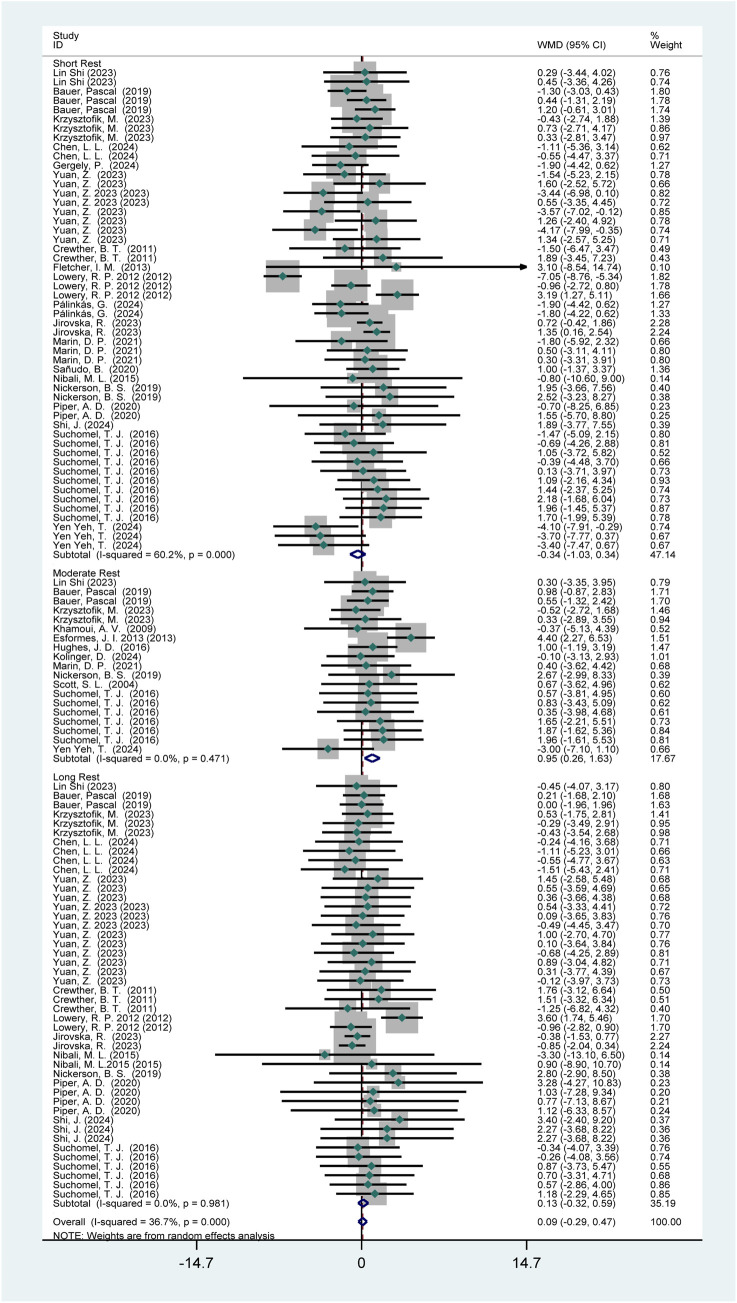
Effects of HIPS on CMJ of male adult athletes.

Twenty-four studies (438 participants) reported the effects of HIPS on vertical jump performance (Figure 11). Meta-analysis showed no significant improvement in CMJ performance (WMD = 0.09; 95% CI: −0.29 to 0.47; *p* = 0.65). Subgroup analysis indicated no significant improvements after short (WMD = −0.34; 95% CI: −1.03 to 0.34; *p* = 0.33) or long intervals (WMD = 0.13; 95% CI: −0.32 to 0.59; *p* = 0.56), but a significant improvement after moderate intervals (WMD = 0.95; 95% CI: 0.26 to 1.63; *p* = 0.01). Moderate heterogeneity was observed (*I* ^2^ = 36.7%).

###### 3.3.3.1.2 Female athletes

**FIGURE 12:**
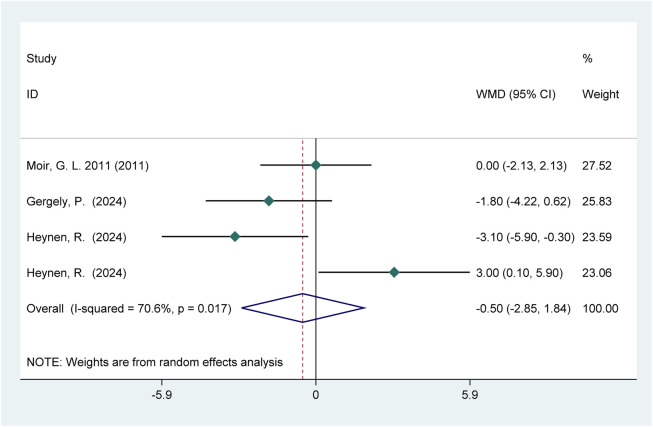
Effects of HIPS on CMJ of female adult athletes.

Three studies (56 participants) reported the effects of HIPS on vertical jump performance (Figure 12). Meta-analysis showed no significant improvement in CMJ performance (WMD = −0.50; 95% CI: −2.85 to 1.84; *p* = 0.673). Moderate heterogeneity was observed (*I* ^2^ = 70.6%).

##### 3.3.3.2 Sprint performance

###### 3.3.3.2.1 Male athletes

**FIGURE 13:**
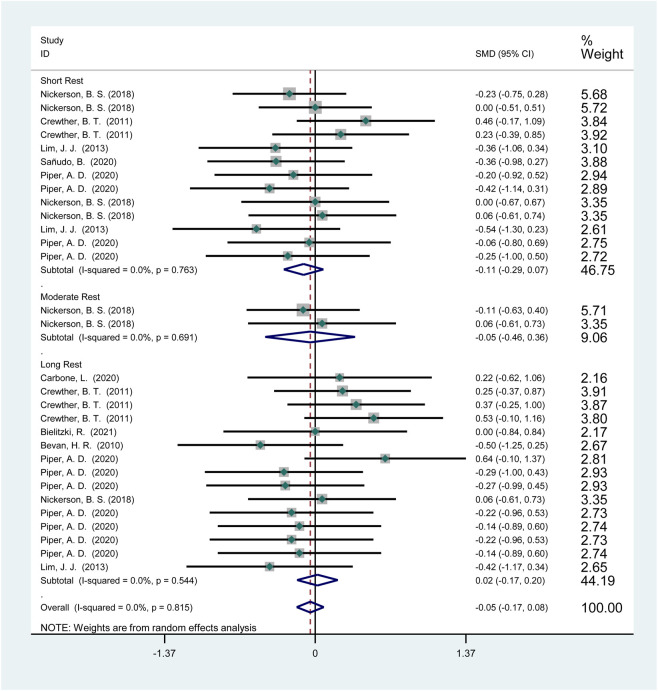
Effects of HIPS on sprint ability of male adult athletes.

Eleven studies (173 participants) reported the effects of HIPS on sprint performance (Figure 13). Meta-analysis revealed no significant improvement in sprint performance (SMD = −0.05; 95% CI: −0.17 to 0.08; *p* = 0.47). Subgroup analysis showed no significant improvements after short (SMD = −0.11; 95% CI: −0.29 to 0.07; *p* = 0.25), moderate (SMD = −0.05; 95% CI: −0.46 to 0.36; *p* = 0.82), or long intervals (SMD = 0.02; 95% CI: −0.17 to 0.20; *p* = 0.84). No heterogeneity was observed (*I* ^2^ = 0%).

### 3.4 Sensitivity analysis

To evaluate the influence of individual studies on the overall effect estimates and heterogeneity, sensitivity analysis was conducted by sequentially excluding each study to assess the robustness of the results. The analysis revealed that the overall effect sizes and their 95% confidence intervals (CIs) remained stable with minimal changes, and the direction of effects was consistently maintained.

Notably, Heynen, R. (2024) was identified as the primary driver of heterogeneity in the CMJ outcomes for female athletes. When included, moderate heterogeneity was observed (*I* ^2^ = 70.6%, *p* = 0.67); after exclusion, heterogeneity significantly decreased (*I* ^2^ = 36.9%, *p* = 0.1). The pooled effect size slightly shifted from −0.50 (95% CI: −2.85 to 1.84) to −1.45 (95% CI: −3.22 to 0.31), while the overall trend remained consistent. Although moderate heterogeneity was observed in some outcomes, a detailed comparison of the studies suggested that the heterogeneity likely originated from differences in recovery intervals. Specifically, Heynen, R. (2024) tested CMJ performance after long recovery intervals, whereas other studies used short intervals. Given the significant influence of recovery time on post-activation effects (PAE), this discrepancy likely contributed to the observed heterogeneity. Additionally, moderate heterogeneity was observed in the CMJ outcomes for low-level athletes and non-Asian athletes, with no significant overall effects and small effect sizes. Subgroup analysis by recovery intervals revealed significant differences or larger effect sizes in short and long intervals, further supporting the hypothesis that recovery time is a key source of heterogeneity.

In conclusion, the influence of individual studies on the overall results was limited, indicating that the findings of this meta-analysis are robust and reliable.

### 3.5 Publication bias

Egger’s test indicated the presence of publication bias for the effect of HIPS on vertical jump performance in high-level adult athletes (*p* = 0.00), while no publication bias was detected for all other outcomes (*p* > 0.05). For outcomes with detected bias, the trim-and-fill method was applied to adjust the effect estimates. No additional studies were identified, and the adjusted effect size remained consistent with the original results (*p* = 0.00), confirming the robustness of the findings and their insensitivity to potential publication bias.

### 3.6 Adverse events

None of the included self-controlled trials reported adverse events related to the intervention. Therefore, no information on adverse events could be extracted from the available literature.

## 4 Discussion

### 4.1 Effects of HIPS on explosive power in high- and low-level adult athletes

This study reveals significant differences in the effects of high-intensity parallel squat (HIPS) on vertical jump performance based on athletic proficiency. High-level adult athletes exhibited a unique dual-window effect: HIPS significantly improved countermovement jump (CMJ) performance during both short (0–4 min, p < 0.05) and moderate recovery intervals (5–8 min, p < 0.05), with the greatest enhancement observed during short intervals. However, no significant improvement was observed after long intervals (>8 min), indicating the disappearance of post-activation effects (PAE). Notably, this acute enhancement effect was highly selective—low-level athletes not only failed to show any improvement but also exhibited a near-significant suppression of performance after short intervals (p = 0.08, WMD = −0.62). This suggests that post-activation potentiation (PAE) is influenced by training background, and optimal recovery intervals may vary based on individual characteristics such as training status and strength levels (Terbalyan et al., 2025). When strength levels are inadequate, the body is more prone to fatigue under resistance, leading to performance declines (Zhu, 2019). Dobbs et al. suggested that recovery intervals shorter than 3 min may negatively impact vertical jump performance (Dobbs et al., 2019). Seitz et al. further noted that experienced athletes exhibit maximal PAE after 6 min, while less experienced athletes show peak enhancement after 9 min, as the former possess superior fatigue resistance and recovery capacity (Seitz et al., 2014a). PAP represents a balance between PAE and fatigue (Mola et al., 2014), and its “window of opportunity” depends on the interplay between these two factors (Mola et al., 2014; Tillin and Bishop, 2009). It is also possible that the generation of PAE involves the activation of the nervous system, which may not be fully optimized in a short period of time, thereby affecting the degree of force output performance (Fernández-Galván et al., 2022). Immediately after HIPS, fatigue accumulates rapidly, dominating the short recovery interval (0–4 min), which explains the performance suppression in low-level athletes and the relatively weaker enhancement in high-level athletes. As recovery time extends, fatigue diminishes, and PAE becomes more pronounced, leading to improved performance. Beyond 8 min, both PAE and fatigue dissipate, and performance returns to baseline levels. This is consistent with the findings of Jiazhe Li (Li et al., 2024) and Yiyan Chen (Chen et al., 2023) in his research, as the results for high- and low-level athletes diverged significantly after short intervals. The fatigue induced by HIPS likely outweighed the PAE during short recovery periods, resulting in reduced CMJ performance (Arabatzi et al., 2014), Therefore, precisely identifying the two “windows of opportunity” for PAE is critical for optimizing performance.

Regarding horizontal jump performance, this study found that HIPS significantly enhanced standing long jump (SLJ) performance in low-level athletes (*p* = 0.01, WMD = 5.79). This result is closely related to the biomechanical characteristics of SLJ and the neuromuscular adaptation potential of low-level athletes. SLJ is a whole-body coordinated movement that relies not only on the explosive power of the lower limb joints (hip, knee, ankle) but also on upper limb swing and core stability (Ashby and Heegaard, 2002), HIPS, as a high-intensity resistance exercise, activates major lower limb muscle groups, potentially optimizing force transmission efficiency and enhancing movement coordination through neuromuscular pre-activation (da Silva et al., 2024). Additionally, low-level athletes, with their lower baseline strength, may exhibit greater sensitivity to HIPS, resulting in more pronounced performance gains (Piper et al., 2020). However, due to the limited number of included studies (2 studies, 26 participants), the optimal recovery interval could not be determined. Existing research has predominantly focused on vertical jumps, with insufficient exploration of SLJ mechanisms, limiting the interpretation of results. For example, da Silva et al. (2024) found that multiple sets of high-load HIPS combined with 6-min intervals activated upper limb muscles but did not establish a causal relationship with SLJ performance. Future studies should expand sample sizes, design controlled experiments with varying recovery intervals, and incorporate kinematic analyses to further clarify the optimal HIPS protocols for SLJ and their underlying physiological mechanisms.

In terms of sprint performance, the results of this study indicate that, unlike vertical jump performance, HIPS did not acutely enhance sprint performance in high-level athletes (*p* = 0.61, WMD = −0.04). This finding aligns with research on the regulatory mechanisms of PAP effects based on strength levels. Studies by Chiu et al. provide critical evidence: when athletes’ squat 1RM/body weight ratio is <2 (indicating low strength levels), their potential to utilize PAP for improving sprint performance is significantly limited (Chiu et al., 2003; Hamada et al., 2000). This suggests that strength may be a key threshold for HIPS-induced improvements in sprint performance, and the lack of enhancement in high-level athletes may stem from their proximity to the physiological limits of neuromuscular adaptation. The regulatory mechanisms of athletic proficiency on PAE can be analyzed from multiple perspectives. First, differences in neuromuscular activation play a role. Duthie et al. demonstrated that high-level athletes achieve greater muscle activation during heavy resistance training, involving mechanisms such as enhanced H-reflex sensitivity and increased myosin regulatory light chain phosphorylation (Duthie et al., 2002). However, this study suggests that such activation advantages may be more applicable to strength-dominant movements, while high-velocity actions like sprinting may trigger compensatory neural inhibition. Although the exact reasons behind the relationship between strength and PAP remain unclear, it has been shown that resistance-trained athletes exhibit greater muscle activation during high-resistance training, influencing the two mechanisms involved in PAP: H-reflex and myosin regulatory light chain phosphorylation (Aagaard et al., 2002). Gullich and Schmidtbleicher found that the gastrocnemius (fast-twitch dominant) exhibits more sustained PAP effects compared to the soleus (slow-twitch dominant), but traditional squat training activates the gastrocnemius to a lesser extent (Gullich and Schmidtbleicher, 1995). This suggests that high-level athletes may require more specific activation strategies to overcome existing adaptation levels in fast-twitch dominant actions like sprinting. Third, energy metabolism characteristics may play a role: the high reliance of sprint performance on the phosphagen system may exacerbate fatigue accumulation in high-level athletes, offsetting the PAE induced by HIPS. In contrast, low-level athletes experience less energy system stress due to lower absolute power output but remain unable to effectively translate PAP effects due to insufficient baseline strength. Notably, this study contrasts with findings by Gullich et al. (Gullich and Schmidtbleicher, 1995) who observed more sustained PAE in elite sprinters compared to student athletes. This discrepancy may arise from differences in task specificity—when the intervention (e.g., squats) does not fully match the muscle activation patterns of the target action (e.g., sprinting), the existing neuromuscular adaptation advantages of high-level athletes may translate into resistance to new stimuli. This provides important insights for future research: the application of HIPS in sprint performance must adhere to the principle of “movement pattern specificity” and design differentiated protocols for athletes of varying proficiency levels.

### 4.2 Effects of HIPS on explosive power in Asian and Non-Asian adult athletes

#### 4.2.1 Vertical jump performance

This study found that HIPS did not significantly improve vertical jump performance (CMJ) in Asian athletes across short, moderate, or long recovery intervals (*p* = 0.86, WMD = 0.05). In contrast, while non-Asian athletes showed no overall acute improvement (*p* = 0.54, WMD = 0.17), they exhibited a significant enhancement after long intervals (>8 min, *p* = 0.01, WMD = 0.86). This discrepancy may stem from regional differences in neuromuscular adaptation mechanisms. The delayed enhancement in non-Asian athletes after long intervals could be attributed to the delayed effects of myosin light-chain phosphorylation (MLCP), potentially due to superior calcium ion reuptake rates or metabolic recovery capacity, allowing PAE to emerge after fatigue subsides (Blazevich and Babault, 2019; Cuenca-Fe et al., 2017). Conversely, Asian athletes may exhibit weaker responses to HIPS due to training backgrounds (e.g., insufficient strength training loads or movement specificity) or genetic factors (e.g., differences in fast-to-slow twitch muscle fiber ratios) (Chiu et al., 2003; Hamada et al., 2000). Additionally, cultural training habits (e.g., greater emphasis on technical training over maximal strength in Asian athletes) may further attenuate the acute effects of HIPS (Chaouachi et al., 2014; Faigenbaum et al., 2009). Notably, although the long-interval improvement in non-Asian athletes was significant, the effect size was small (WMD = 0.86), suggesting a need to balance time costs and benefits in practical applications. Future studies should incorporate electromyography (EMG) and biomechanical analyses to clarify the physiological basis of these regional differences.

#### 4.2.2 Horizontal jump performance

Non-Asian adult athletes demonstrated significant improvements in standing long jump (SLJ) performance following HIPS (*p* = 0.02, WMD = 4.23). However, due to the limited number of included studies (4 studies, 60 participants), the optimal recovery interval remains unclear. SLJ, as a whole-body coordinated movement, may benefit from HIPS through enhanced force transmission efficiency across the hip, knee, and ankle joints (Ashby and Heegaard, 2002). Additionally, the compensatory role of arm swing in SLJ may be indirectly enhanced by HIPS, as pre-activation of upper limb muscles could optimize overall movement coordination through neural coupling mechanisms. However, existing research has predominantly focused on vertical jumps, limiting the interpretation of SLJ results. For example, da Silva et al. (2024) found that multiple sets of high-load HIPS combined with 6-min intervals activated upper limb muscles but did not establish a causal relationship with SLJ performance. Future studies should design targeted experiments with controlled recovery intervals (e.g., 4–12 min), incorporate kinematic analyses, and expand sample sizes to enhance the generalizability of findings.

#### 4.2.3 Sprint performance

Despite the partial benefits of HIPS on vertical and horizontal jump performance in non-Asian athletes, no acute improvement in sprint performance was observed (*p* = 0.36, WMD = −0.06). This contradiction highlights the influence of movement specificity on PAP effects. Sprinting, as a multi-planar, high-frequency movement, relies on rapid energy supply from the phosphagen system and the explosive power of hip flexor muscles (Dinyer et al., 2021), Traditional HIPS, which primarily involves sagittal plane knee extension, may not adequately match the mechanical demands of sprinting. Furthermore, fatigue induced by HIPS may offset neural gains, particularly in high-level athletes whose output power approaches physiological limits, making them more susceptible to energy depletion (Hamada et al., 2000; Gullich and Schmidtbleicher, 1995). For instance, Piper et al. (Piper et al., 2020) observed a decline in sprint performance within 20 s post-HIPS, with no significant improvement even after 4–8 min of recovery, suggesting the need to optimize load strategies (e.g., reducing intensity to <75% 1RM) or incorporating eccentric overload training. Future research should explore combined interventions of HIPS and sprint-specific exercises (e.g., weighted acceleration runs) and include metabolic markers (e.g., blood lactate) to comprehensively assess the dynamic balance between fatigue and PAE. It should be noted that “region” serves only as a crude proxy; the observed disparities may stem from unmeasured confounders such as specific training practices, genetic background, or dietary habits.

### 4.3 Effects of HIPS on explosive power in male and female adult athletes

#### 4.3.1 Vertical jump performance

This study revealed sex-specific effects of HIPS on vertical jump performance, with significant differences in mechanisms between male and female athletes. Analysis of gender-related variables closely associated with performance showed that male athletes exhibited an acute enhancement window during moderate recovery intervals (4–8 min, *p* = 0.01, WMD = 0.95), while female athletes showed no HIPS-induced improvements (*p* = 0.64, WMD = −0.45). This gender disparity can be explained through neuromuscular adaptation characteristics and fatigue metabolism mechanisms. This phenomenon is directly related to male muscle mass advantages—higher testosterone levels enhance fast-twitch fiber recruitment efficiency, enabling rapid remodeling of phosphorylated regulatory light chains within 4–8 min (Hicks et al., 2001). However, fatigue induced by HIPS may outweigh PAE, resulting in no CMJ improvement. Female Fatigue Sensitivity: Notably, in the female subgroup the wide confidence interval crosses the null value ((WMD = −0.50; 95% CI: −2.85 to 1.84), indicating not only an absence of effect but also insufficient precision. Female athletes experience more pronounced fatigue accumulation under the same HIPS protocol, with mechanisms involving multiple dimensions. Gergely et al. also reported no acute CMJ enhancement in female athletes post-HIPS, attributing this to excessive fatigue from high repetition volumes, a response observed in both genders (Pálinkás et al., 2024). Studies have shown that knee extensor fatigue reduces jump height by approximately 14%, while knee flexor fatigue reduces it by 6% (Rodacki et al., 2002). Overall, acute neuromuscular performance changes post-HIPS are similar between males and females (Dinyer et al., 2021; Keller et al., 2022). However, males exhibit greater reductions in muscle oxygen consumption and fatigue under the same intensity, potentially due to higher absolute loads and total energy expenditure (Pálinkás et al., 2024). These findings have important implications for training: male athletes can utilize HIPS with 4–8 min intervals to achieve peak vertical power outpu.

#### 4.3.2 Sprint performance

This study identified sex-specific effects of HIPS on sprint performance: male athletes showed no acute improvements across short (0–4 min), moderate (4–8 min), or long (>8 min) intervals, contrasting sharply with gender differences in vertical jump performance. This disparity can be attributed to three key factors: At the first, interaction Between Movement Patterns and Gender Adaptation: The “movement specificity principle” proposed by Suarez-Arrones et al. is extended in this study: male athletes, despite benefiting from sagittal plane-dominant HIPS interventions like squats, struggle to transfer these gains to multi-planar, high-frequency sprinting movements. Gender differences further amplify this effect—male-dominant muscle groups (e.g., quadriceps) are highly activated in traditional HIPS protocols, but this “path dependency” limits their adaptation potential in asymmetric explosive actions (Suarez-Arrones et al., 2020), rendering HIPS ineffective for subsequent sprint performance. Second, Insufficient Muscle Fiber Stimulation:Differences in PAPE effects observed in HIPS groups may stem from insufficient activation of fast-twitch fibers. Suarez-Arrones et al. reported significant sprint performance improvements following flywheel eccentric training, which induces greater stretch reflexes during the transition between eccentric and concentric phases, enhancing subsequent concentric performance more effectively than traditional resistance training (Wilson et al., 2013). One last point, Influence of Recovery Intervals: Recovery time significantly impacts acute performance. Lim et al. found no PAP effect on 10 m and 20 m sprint performance, likely due to their choice of a 4-min recovery interval, which may have negatively influenced results (Lim and Kong, 2013), Similarly, Till et al. support this view (Till and Cooke, 2009). In contrast, some studies report positive PAP effects on sprint performance (Chatzopoulos et al., 2007; Linder et al., 2010). though these findings are uncertain due to non-randomized experimental designs. For example, Piper et al. observed maximal sprint performance declines 20 s post-HIPS, with improvements only after 4–8 min, suggesting that recovery interval timing may explain conflicting conclusions across studies (Piper et al., 2020), So the interval may be the reason why Piper and Lim et al. ‘s findings contradict each other.

## 5 Conclusion

This study demonstrates that high-intensity parallel squat (HIPS) elicits significant differences in the acute effects on lower limb explosive power in adult athletes: high-level athletes exhibit significant improvements in vertical jump performance during short (0–4 min) and moderate (5–8 min) recovery intervals, while low-level athletes benefit only in horizontal jump performance. Non-Asian athletes show superior vertical jump performance after long intervals (>8 min), and male athletes outperform females during moderate recovery intervals. HIPS does not significantly enhance sprint performance.

## 6 Practical implications

Individualized Protocol: Athletes with high proficiency (squat 1RM/weight ≥2) can combine short/medium recovery intervals of HIPS warm-up to optimize vertical jump performance; athletes with low proficiency need to prioritize enhancing their basic strength before considering using HIPS for activation to improve acute exercise performance.

Gender and Region Adaptation: Males are recommended to rest for 5–8 min after intervention activation and then proceed with training. Non-Asian athletes can attempt a long interval (>8 min) strategy.

## 7 Research limitation

The “Asian/non-Asian” dichotomy employed in this study may obscure physiologic or training-related variables that are more pertinent than geographic origin *per se*, and the resulting estimates should therefore be interpreted with caution. Sample Size and Heterogeneity: Some subgroups had small sample sizes (e.g., only 3 studies for female CMJ and 2 studies for SLJ), a circumstance that amplifies sampling error and, consequently, attenuates the certainty of the corresponding conclusions. Additionally, heterogeneity in HIPS protocols (sets/repetitions) and participant characteristics (sport type, age) across studies may affect the stability of the results. Additionally, heterogeneity in the high-intensity pre-conditioning protocols (e.g., number of sets and repetitions) represents a potential source of variability, even though the relative intensity has been standardized. Language bias may exist because only English-language publications were included. Despite evaluation and adjustment with Egger’s test and the trim-and-fill method, publication bias was still detected for some outcomes (e.g., CMJ in high-level athletes), which could limit the generalis ability of the findings.
