# Supplementary material for: Personalized warm-up strategies for adult athletes: a meta-analysis based on athletic level, gender, and region
Source: Front Physiol. 2025 Dec 4;16:1706583. doi: 10.3389/fphys.2025.1706583 (PMC12711518; doi:10.3389/fphys.2025.1706583)
Supplement: Supplementary file 3 [file Table1.docx]

Table 1. Basic characteristics of the included studies

| **Serial** | **Author (Year)** | **Sample** | **Load (Sets×Reps×%1RM)** | **Intervals (min)** | **Gender** | **Region** | **Index** |
| --- | --- | --- | --- | --- | --- | --- | --- |
| 1 | Lin Shi 2023 | 13 | 1×3×85% | 0.5, 3, 6, 9 | Male | Asia | CMJ |
| 2 | Atalag et al.2021 | 19 | 1×3×90% | 3 | All | Other | CMJ, RSI |
| 3 | Matthews et al.04 | 20 | 1×5×85% | 10 | Male | Other | 20m |
| 4 | Bauer 2019 | 60 | 1×5×60%; 1×4×90% | 0.25–11 | Male | Asia | CMJ |
| 5 | Krzysztofik 2023 | 16 | 3×3×85% | 3, 6, 9 | Male | Other | CMJ |
| 6 | Atalağ et al.2020 | 17 | 1×3×90% | 8 | All | Other | CMJ, 20y, 40y |
| 7 | Krzysztofik 2023 | 15 | 1×2×60%; 3×3×85% | 2–10 | Male | Other | CMJ |
| 8 | Boyd et al.2014 | 10 | 1×3×90% | 2, 5, 8, 11 | Male | Other | CMJ |
| 9 | Chen et al.2024 | 18 | 1×3×93% | 0.25–20 | Male | Other | CMJ |
| 10 | Carbone et al.2020 | 17 | 3×3×85% | 8 | Male | Other | 5m,10m |
| 11 | Moir et al.2011 | 11 | 1×12×37%; 1×3×90% | 2 | Female | Asia | CMJ |
| 12 | Urbanski et al.2023 | 12 | 1×3×90% | 5 | unknow | Other | CMJ, SLJ |
| 13 | Crum et al.2012 | 20 | 3×1×50%; 3×1×65% | 3,5,10,15 | Male | Other | CMJ |
| 14 | Nickerson et al.2018 | 12 | 1×3×85% | 1,4,7,10 | Male | Other | 10m,20m |
| 15 | Gergely et al.2024 | 29 | 5×3×85% | 0 | Male | Other | CMJ |
| 16 | Gergely et al.2024 | 29 | 5×3×85% | 0 | Female | Other | CMJ |
| 17 | Yuan et al.2023 | 22 | 2×5×85% | 4,8,12,16 | Male | Asia | CMJ, PPO |
| 18 | Do Carmo et al.2021 | 12 | 1×5×85% | 4 | Male | Other | CMJ |
| 19 | Crewther et al.2011 | 9 | 1×3×90% | 4,8,12,16 | Male | Other | CMJ,5m,10m |
| 20 | Khamoui et al.2009 | 16 | 5×3×85% | 5 | Male | Other | CMJ |
| 21 | Faller et al.2023 | 14 | 1×3×90% | 0.75 | Male | Other | CMJ |
| 22 | Seitz et al.2014 | 13 | 1×3×90% | 7 | unknow | Other | 20m |
| 23 | Fletcher et al.2013 | 16 | 1×2×90% | 4 | Male | Other | CMJ,SJ,DJ |
| 24 | Bielitzki et al.2021 | 18 | 1×3×91% | 8 | Male | Other | 5m,10m |
| 25 | Comyns et al.2010 | 11 | 1×3×91% | 4 | Male | Other | 10,20,30m |
| 26 | Esformes et al.2013 | 27 | 1×3×91% | 5 | Male | Other | CMJ |
| 27 | Lowery et al.2012 | 13 | Multi-load | 0–12 | Male | Other | CMJ, PPO |
| 28 | Heynen et al.2024 | 16 | 3×3×85% | 2min, 2h | Female | Other | CMJ, SJ |
| **Serial** | **Author (Year)** | **Sample** | **Load (Sets×Reps×%1RM)** | **Intervals (min)** | **Gender** | **Region** | **Index** |
| 29 | McCann et al.2010 | 16 | 1×5×85% | 4,5 | All | Other | CMJ |
| 30 | Hornikel et al.2023 | 13 | 1×4×75% | 4 | unknow | Other | CMJ |
| 31 | Lim et al.2013 | 12 | 1×3×90% | 4 | Male | Other | 10,20,30m |
| 32 | Bevan et al.2010 | 16 | 1×3×91% | 8 | Male | Other | 5m,10m |
| 33 | Santos et al.2023 | 14 | 3×3×90% | 6 | Female | Other | CMJ, SLJ |
| 34 | Pálinkás et al.2024 | 29 | 5×3×85% | 0 | Male | Other | CMJ |
| 35 | Hughes et al.2016 | 16 | 1×3×91% | 6 | Male | Other | CMJ |
| 36 | Kolinger et al.2024 | 12 | 1×3×90% | 5,8,11 | Male | Other | CMJ |
| 37 | Villalon et al.2022 | 12 | 1×3×90% | 8 | unknow | Asia | CMJ |
| 38 | West et al.2013 | 36 | 1×3×87% | 8 | unknow | Other | CMJ (PPO) |
| 39 | Jirovska et al.2023 | 13 | 1×3×85% | 0.5,4,8,12 | Male | Other | CMJ |
| 40 | Márquez et al.2023 | 17 | 1×3×80% | 1,4 | Male | Other | CMJ, DJ |
| 41 | Marin et al.2021 | 9 | 3×3×85% | 2,4,6,8 | Male | Other | CMJ |
| 42 | Sañudo et al.2020 | 28 | 1×3×90% | 4 | unknow | Other | CMJ,10m |
| 43 | Evetovich et al.2015 | 20 | 1×3×80% | 8 | All | Other | CMJ, SLJ |
| 44 | Evetovich et al.2015 | 11 | 1×3×80% | 8 | Male | Other | CMJ |
| 45 | Montalvo et al.2021 | 14 | 1×5×85% | 3 | All | Other | CMJ |
| 46 | Nibali et al.2015 | 8 | 1×5×85% | 4,8,12 | Male | Other | CMJ |
| 47 | Nickerson et al.2019 | 12 | 1×3×85% | 1,4,7,10 | Male | Other | CMJ |
| 48 | Hester et al.2017 | 14 | 1×5×80% | 1,3,5,10 | Male | Other | CMJ |
| 49 | Piper et al.2020 | 13 | 3×5×87% | 0.33–20 | Male | Other | CMJ,10,20m |
| 50 | Mina et al.2019 | 15 | 1×3×85% | 0.5,4,8,12 | Male | Other | CMJ |
| 51 | Scott et al.2004 | 19 | 1×5×85% | 5 | Male | Other | CMJ, SLJ |
| 52 | Shi et al.2024 | 13 | 5×1×90% | 0,4,8,12 | Male | Asia | CMJ |
| 53 | Suchomel et al.2016 | 16 | 1×2×90% | 1–10 | Male | Other | CMJ |
| 54 | Sue et al.2016 | 9 | 1×5×85% | 2,6,10,14,18 | Female | Other | SLJ |
| 55 | Villalon et al.2020 | 11 | 1×3×90% | 8 | unknow | Other | CMJ |
| 56 | Yen Yeh et al.2024 | 17 | 3×3×85% | 0,2,4,6 | All | Other | CMJ |
| 57 | Zheng et al.2024 | 20 | 1×15×30% | 0,3,6,9,12 | Male | Asia | CMJ |
| 58 | Guo et al.2018 | 13 | 1×3×90% | 3,6,9,12 | Male | Asia | CMJ |
