# Supplementary material for: Personalized warm-up strategies for adult athletes: a meta-analysis based on athletic level, gender, and region
Source: Front Physiol. 2025 Dec 4;16:1706583. doi: 10.3389/fphys.2025.1706583 (PMC12711518; doi:10.3389/fphys.2025.1706583)
Supplement: Supplementary file 4 [file Table4.docx]

| Database | Web of Science (all database) | |
| --- | --- | --- |
| Search Date | July 4th, 2025, 2025 | |
| Search Period | From the inception of database to July 4th, 2025, 2025 | |
| No. | Search strategy | Literatures retrieved |
| #1 | TS=("Barbell Squat" or "Back Squat" or "Low Bar Squat" or "High Bar Squat") | **1067** |
| #2 | TS=("Acute responses" or "Acute Effects" or "post-activation potentiation" or "post-activation performance enhancement" or "Postactivation Potentiation" or "postactivation performance enhancement") | **7440** |
| #3 | TS=("Jump" or "Counter Movement Jump" or "Vertical Jump" or "Squat Jump" or "Drop Jump" or "Abalakov Jump" or "Bosco Repeat Vertical Jump" or "Standing long jump" or "Standing broad jump" or "Power" or "Maximum Output" or "Maximum Output" or "Peak Strength" or "Peak Output" or "1RM" or "Sprint" or "Speed" or "5m" or "10m" or "20m" or "30m" or "5 meters" or "10 meters" or "20 meters" or "30 meters") | **2620010** |
| #4 | #3 AND #2 AND #1 | **128** |

| Database | PubMed | |
| --- | --- | --- |
| Search Date | July 4th, 2025 | |
| Search Period | From the inception of database to July 4th, 2025 | |
| No. | Search strategy | Literatures retrieved |
| #1 | "Barbell Squat"[Title/Abstract] OR "Back Squat"[Title/Abstract] OR "Low Bar Squat"[Title/Abstract] OR "High Bar Squat"[Title/Abstract] | **958** |
| #2 | "Acute responses"[Title/Abstract] OR "Acute Effects"[Title/Abstract] OR "post-activation potentiation"[Title/Abstract] OR "post-activation performance enhancement"[Title/Abstract] OR "Postactivation Potentiation"[Title/Abstract] OR "postactivation performance enhancement"[Title/Abstract] | **16581** |
| #3 | "Jump" or "Counter Movement Jump" or "Vertical Jump" or "Squat Jump" or "Drop Jump" or "Abalakov Jump" or "Bosco Repeat Vertical Jump" or "Standing long jump" or "Standing broad jump" or "Power" or "Maximum Output" or "Maximum Output" or "Peak Strength" or "Peak Output" or "1RM" or "Sprint" or "Speed" or "5m" or "10m" or "20m" or "30m" or "5 meters" or "10 meters" or "20 meters" or "30 meters" | **848434** |
| #4 | #3 AND #2 AND #1 | **116** |

| Database | EBSCOhost | |
| --- | --- | --- |
| Search Date | July 4th, 2025 | |
| Search Period | From the inception of database to July 4th, 2025 | |
| No. | Search strategy | Literatures retrieved |
| #1 | AB "Barbell Squat" or "Back Squat" or "Low Bar Squat" or "High Bar Squat" | **1622** |
| #2 | AB "Acute responses" or "Acute Effects" or "post-activation potentiation" or "post-activation performance enhancement" or "Postactivation Potentiation" or "postactivation performance enhancement" | **13583** |
| #3 | AB"Jump" or "Counter Movement Jump" or "Vertical Jump" or "Squat Jump" or "Drop Jump" or "Abalakov Jump" or "Bosco Repeat Vertical Jump" or "Standing long jump" or "Standing broad jump" or "Power" or "Maximum Output" or "Maximum Output" or "Peak Strength" or "Peak Output" or "1RM" or "Sprint" or "Speed" or "5m" or "10m" or "20m" or "30m" or "5 meters" or "10 meters" or "20 meters" or "30 meters" | **2570779** |
| #4 | #3 AND #2 AND #1 | **107** |

| Database | Scopus | |
| --- | --- | --- |
| Search Date | July 4th, 2025 | |
| Search Period | From the inception of database to July 4th, 2025 | |
| No. | Search strategy | Literatures retrieved |
| #1 | TITLE-ABS-KEY ( "Barbell Squat" OR "Back Squat" OR "Low Bar Squat" OR "High Bar Squat" ) | **1426** |
| #2 | TITLE-ABS-KEY ( "Acute responses" OR "Acute Effects" OR "post-activation potentiation" OR "post-activation performance enhancement" OR "Postactivation Potentiation" OR "postactivation performance enhancement" ) | **27470** |
| #3 | TITLE-ABS-KEY ( "Jump" OR "Counter Movement Jump" OR "Vertical Jump" OR "Squat Jump" OR "Drop Jump" OR "Abalakov Jump" OR "Bosco Repeat Vertical Jump" OR "Standing long jump" OR "Standing broad jump" OR "Power" OR "Maximum Output" OR "Maximum Output" OR "Peak Strength" OR "Peak Output" OR "1RM" OR "Sprint" OR "Speed" OR "5m" OR "10m" OR "20m" OR "30m" OR "5 meters" OR "10 meters" OR "20 meters" OR "30 meters" ) | **6,637,948** |
| #4 | #3 AND #2 AND #1 | **199** |

| Database | Cochrane | |
| --- | --- | --- |
| Search Date | July 4th, 2025 | |
| Search Period | From the inception of database to July 4th, 2025 | |
| No. | Search strategy | Literatures retrieved |
| #1 | "Barbell Squat" or "Back Squat" or "Low Bar Squat" or "High Bar Squat" in Title Abstract Keyword | **483** |
| #2 | "Acute responses" or "Acute Effects" or "post-activation potentiation" or "post-activation performance enhancement" or "Postactivation Potentiation" or "postactivation performance enhancement" in Title Abstract Keyword | **7591** |
| #3 | "Jump" or "Counter Movement Jump" or "Vertical Jump" or "Squat Jump" or "Drop Jump" or "Abalakov Jump" or "Bosco Repeat Vertical Jump" or "Standing long jump" or "Standing broad jump" or "Power" or "Maximum Output" or "Maximum Output" or "Peak Strength" or "Peak Output" or "1RM" or "Sprint" or "Speed" or "5m" or "10m" or "20m" or "30m" or "5 meters" or "10 meters" or "20 meters" or "30 meters" in Title Abstract Keyword | **924** |
| #4 | #3 AND #2 AND #1 | **75** |

| Database | Embase | |
| --- | --- | --- |
| Search Date | July 4th, 2025 | |
| Search Period | From the inception of database to July 4th, 2025 | |
| No. | Search strategy | Literatures retrieved |
| #1 | 'barbell squat':ab,ti OR 'back squat':ab,ti OR 'low bar squat':ab,ti OR 'high bar squat':ab,ti | 891 |
| #2 | 'acute responses':ab,ti OR 'acute effects':ab,ti OR 'post-activation potentiation':ab,ti OR 'post-activation performance enhancement':ab,ti OR 'postactivation potentiation':ab,ti OR 'postactivation performance enhancement':ab,ti | 21573 |
| #3 | 'jump':ab,ti OR 'counter movement jump':ab,ti OR 'vertical jump':ab,ti OR 'squat jump':ab,ti OR 'drop jump':ab,ti OR 'abalakov jump':ab,ti OR 'bosco repeat vertical jump':ab,ti OR 'standing long jump':ab,ti OR 'standing broad jump':ab,ti OR 'power':ab,ti OR 'maximum output':ab,ti OR 'peak strength':ab,ti OR 'peak output':ab,ti OR '1rm':ab,ti OR 'sprint':ab,ti OR 'speed':ab,ti OR '5m':ab,ti OR '10m':ab,ti OR '20m':ab,ti OR '30m':ab,ti OR '5 meters':ab,ti OR '10 meters':ab,ti OR '20 meters':ab,ti OR '30 meters':ab,ti | 896767 |
| #4 | #3 AND #2 AND #1 | 98 |
